# Fatty Acid Profile in the Liver of Mice with Early- and Late-Onset Forms of Huntington’s Disease

**DOI:** 10.3390/ijms26157304

**Published:** 2025-07-28

**Authors:** Magdalena Gregorczyk, Adriana Mika, Tomasz Śledziński, Marta Tomczyk, Iwona Rybakowska

**Affiliations:** 1 Department of Biochemistry and Clinical Physiology, Medical University of Gdansk, 80-210 Gdansk, Poland; magdalena.gregorczyk@gumed.edu.pl; 2 Department of Environmental Analysis, Faculty of Chemistry, University of Gdansk, 80-309 Gdansk, Poland; adriana.mika@gumed.edu.pl; 3 Department of Pharmaceutical Biochemistry, Medical University of Gdansk, 80-210 Gdansk, Poland; tomasz.sledzinski@gumed.edu.pl; 4 Department of Biochemistry, Medical University of Gdansk, 80-210 Gdansk, Poland; marta.tomczyk@gumed.edu.pl

**Keywords:** Huntington’s disease, liver, fatty acids, IL1-α

## Abstract

Huntington’s disease (HD) is characterized by progressive neurodegeneration, but increasing evidence points to multisystemic involvement, including early hepatic steatosis in pediatric HD. Therefore, it is important to consider systemic alterations, particularly in liver lipid metabolism. In this study, we analyzed fatty acid (FA) profiles in two symptomatic HD mouse models: 2-month-old *R6/2* mice representing early-onset HD and 22-month-old *Hdh^Q150/Q150^* (*Hdh*) mice representing late-onset HD, along with age-matched wild-type (*WT*) controls. FA composition in liver tissue was assessed by gas chromatography–mass spectrometry (GC–MS). In *R6/2* mice, we observed increased levels of total iso-branched chain, monounsaturated, and n-6 polyunsaturated FAs compared to WT. In contrast, only a few FA species showed reduced concentrations in *Hdh* mice. Overall, our results indicate that *R6/2* mice exhibit more pronounced alterations in hepatic FA profiles than *Hdh* mice, suggesting that early-onset HD may be associated with more severe peripheral metabolic dysregulation.

## 1. Introduction

Huntington’s disease (HD) is an autosomal dominant neurodegenerative disorder whose phenotype includes chorea movements, incoordination, behavioral disturbances, and psychiatric symptoms [1,2,3]. HD is caused by a CAG trinucleotide expansion in the huntingtin (HTT) gene, resulting in the formation of a variable-length polyglutamine strand at the N-terminus [1]. The average age of onset of the disease is around 40 years, but there are also juvenile forms that manifest before the age of 20 (5–10% of cases) and late onset of the disease after the age of 50 (20%). Unaffected individuals have a range of 6–35 CAG repeats in the HTT gene, while individuals with HD have 36–121 CAG repeats [4]. With more than 80 CAG repeats in the HTT gene, the disease appears in the first year of life [5,6]. The presence of normally functioning individuals aged 75–90 years with CAG expansions in the 36–39 range suggests that some people with small HD expansions can survive into old age without symptoms [7]. Pediatric HD contributes to an atypical, disabling, and life-shortening phenotype compared to adult HD [5]. In adult HD, the greatest CAG expansion is observed in the striatum and cerebral cortex [6]. In humans with HD, the liver exhibited the highest level of CAG expansion among the periphery tissues [8]. However, postmortem histologic examinations of the liver of adult HD patients have not revealed any morphologic changes. Changes are observed in childhood HD, where an increase in liver volume and steatosis are noted [6]. It, therefore, seems important to compare the metabolic changes that occur in early- and late-onset HD at the molecular level.

Lipid membranes might contribute to the spontaneous appearance of mHTT aggregates in the brain of *R6/2* mice [9], and likely the same situation occurs in the liver. The global genomic organization and gene expression are similar between humans and mice. Like humans, mice have basic neural circuits and types of neuronal cells that are selectively affected in HD along with a rich behavioral repertoire associated with specific neuronal dysfunction and degeneration [10,11]. The liver is an important site of systemic energy metabolism, and the numerous metabolic pathways are regulated in this tissue hormonally and neuronally as well as by nutritional status [12]. Mitochondrial dysfunction is observed in the liver of HD patients [13,14], which can alter lipid metabolism and other metabolic pathways. Fatty acids (FAs) play an important role in the body: they have structural functions as components of phospholipids that build cell membranes, they serve as storage materials in cells, and their derivatives are involved in cell signaling [15]. Steatosis is an excessive ectopic accumulation of lipids in the liver that causes lipotoxicity and leads to harmful metabolic consequences such as endoplasmic reticulum stress and inflammation [16,17,18,19]. Linoleic acid (LA, 18:2n-6) is a precursor of gamma-linolenic acid (GLA, 18:3n-6), dihomo-gamma-linolenic acid (DGLA, 20:3n-6), and arachidonic acid (ARA, 20:4n-6) [20]. ARA is a precursor of several potent pro-inflammatory messengers, including well-described prostaglandins and leukotrienes [21]. HD is associated with increased inflammation, as evidenced by elevated levels of circulating proinflammatory interleukins [22]. Together with HD-associated hepatic steatosis, this may indicate increased inflammation in the liver [23]. However, the relationship between HD and liver inflammation and FA composition has not yet been investigated.

The pathological mechanisms of HD are still being investigated, and research on transgenic mice is making it possible to discover the molecular mechanisms of disease progression. Considering the occurrence of hepatic steatosis in the course of early-onset HD, and the fact that FA are substrates for the synthesis of various compounds with pro-inflammatory and anti-inflammatory properties, in the present study, we investigated FA composition and levels in the liver of two HD mouse models, *R6/2* and *Hdh^Q150/Q150^* (*Hdh*), representing the early and late onset of HD, respectively.

## 2. Results

### 2.1. Lipid Concentration in the Liver

**Figure 1:**
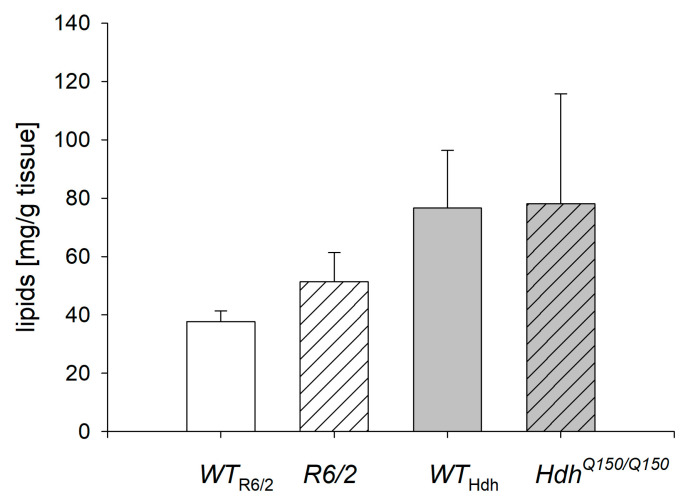
Lipid concentration in the liver of mice with Huntington’s disease *R6/2* and *Hdh^Q150/Q150^* compared to *WT* mice of the corresponding age (*WT*_R6/2_—wild type for *R6/2*; *WT*_Hdh_—wild type for *Hdh^Q150/Q150^*). Data presented as mean ± SEM.

Although there was a trend towards higher lipid content in *R6/2* mice compared to their wild-type (*WT*) controls, there were no statistical differences in total lipid concentration in the livers of *R6/2* and *Hdh^Q150/Q150^* mice and their corresponding *WT* controls (Figure 1).

### 2.2. Fatty Acid Composition in the Liver

**Figure 2:**
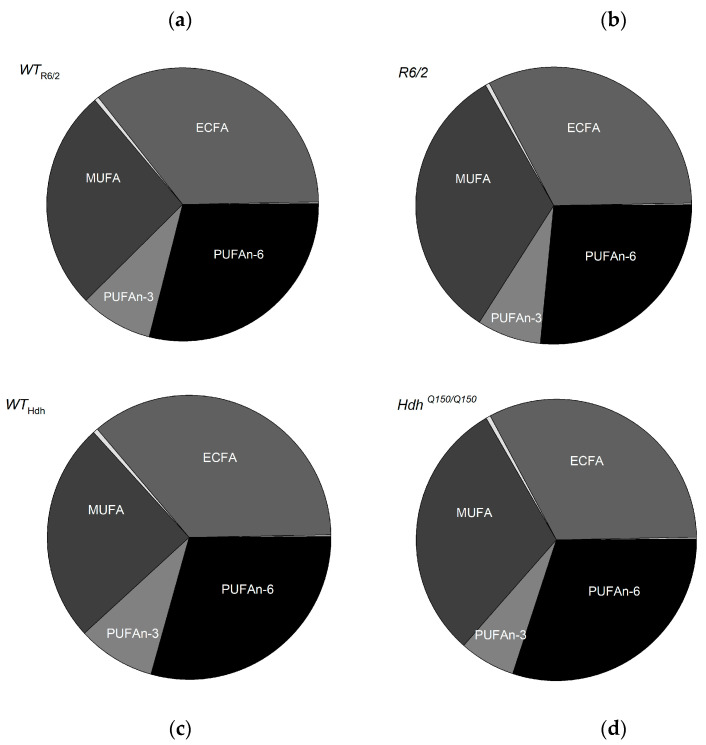
Total concentrations of FA groups: iso BCFA, BCFA, ECFA, OCFA, MUFA, PUFAn-3, and PUFAn-6 in liver of (**a**) *WT*_R6/2_, (**b**) *R6/2*, (**c**) *WT*_Hdh_, and (**d**) *Hdh^Q150/Q150^* mice.

Among the FAs analyzed with a length between 12–30 carbon atoms (saturated, monounsaturated, and polyunsaturated), significantly more differences in their profile were observed in *R6/2* mice, an early-onset form of the disease. Higher levels of iso16:0, iso17:0, 18:1, 19:1, 20:1, 20:4n-3 (ETA), 20:5n-3 (EPA), 18:2n-6 (LA), 20:2n-6 (EDA), 20:3n-6 (DGLA), 20:4n-6 (ARA), and 22:4n-6 (AdA) were found in the liver of *R6/2* mice compared to *WT* (Table 1). Overall, the content of iso-branched-chain FA (isoBCFA), monounsaturated FA (MUFA), and n-6 polyunsaturated fatty acids (n-6 PUFA) was higher in the livers of *R6/2* mice than in those of *WT* (Table 1, Figure 2a,b).

Of the FAs tested in the livers of *Hdh^Q150/Q150^* mice, only the levels of EPA, 22:5n-3 (DPA), and AdA were decreased in *Hdh^Q150/Q150^* mice compared to *WT* (Table 2, Figure 2c,d). This suggests different mechanisms of regulation of FA metabolism in *R6/2* and *Hdh^Q150/Q150^* mice with early and late disease onset, respectively.

### 2.3. IL-1α Level in the Liver

**Figure 3:**
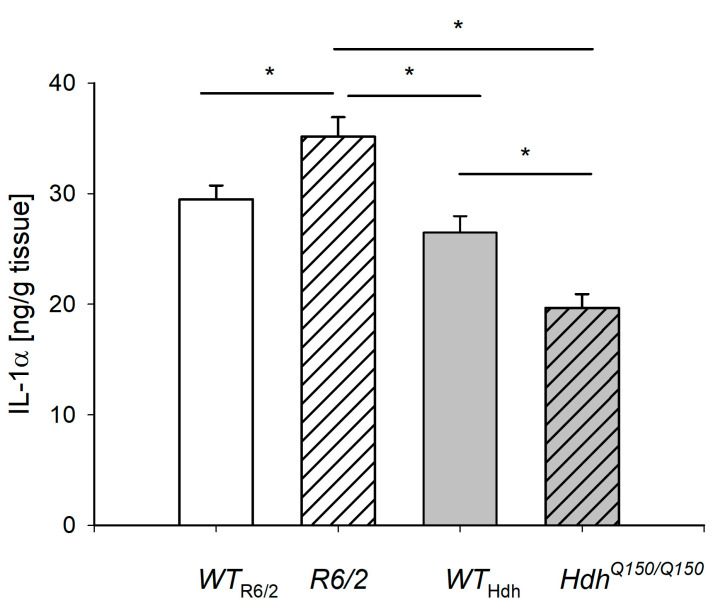
Level of IL-1α in the liver of Huntington’s disease mouse models. *R6/2* and *Hdh^Q150/Q150^* mouse models were compared to the *WT* mice (*WT*_R6/2_-wild type for *R6/2* and *WT*_Hdh_-wild type for *Hdh^Q150/Q150^*). Data presented as mean ± SEM; * *p* < 0.05.

To assess inflammation, the IL-1α level was measured (Figure 3). IL-1α level increased in *R6/2* mice and decreased in *Hdh^Q150/Q150^* mice, indicating different regulation of the inflammatory process in early- and late-onset HD.

## 3. Discussion

Although HD primarily affects the brain, increasing evidence points to systemic metabolic alterations, including those occurring in the liver. Our previous studies have demonstrated that metabolic dysregulation in HD extends beyond the central nervous system, impacting peripheral organs such as the heart and skeletal muscles [24,25,26,27]. In the mouse model of Huntington’s disease *BACHD* presenting slow progression of the disease, which carries the full-length human *HTT* gene, increased levels of inflammatory cytokines IL-12p70 and TNFα were found in the liver [28]. *R6/2* and *Hdh^Q150^* mice are more susceptible than *WT* mice to lipopolysaccharide-evoked systemic inflammation and produced more proinflammatory cytokines in the brain [29].

In this study we highlighted a significant change in the FA profile in the livers of mice with early-onset HD (*R6/2*), which may be associated with the development of hepatic steatosis and liver inflammation. In contrast, few changes in FA levels were found in *Hdh^Q150/Q150^* mice with late-onset HD compared to *WT*. The lipid content in *R6/2* tended to be increased compared to *WT*, but this trend was not significant. This could indicate the early stage of development of liver steatosis but does not fully reflect the changes observed in early-onset HD in humans. Nonetheless, this trend could partially explain the fact that some FAs whose levels were significantly altered in *R6/2* mice were elevated. Another finding suggesting an early stage in the development of hepatic steatosis is a significant increase in 18:1, a major component of TAG [30]. In the *Hdh^Q150/Q150^* mice, there were no significant changes in either total liver lipids or 18:1.

There are very few data on the association of FA metabolism with HD. However, there is evidence that mutant huntingtin (mHTT) affects SREBPs and thus may limit de novo cholesterol and FA synthesis in the brain [31]. However, our results from the liver do not indicate a limitation of de novo SFA or MUFA synthesis because some representatives of these FA groups are present in higher amounts in the liver of *R6/2* mice compared to *WT*. We can only speculate that some brain–liver axis may be responsible for the FA alterations in the liver. It has been shown that mutation in the gene encoding huntingtin leads to increased formation of membrane structures within cells [32,33]. The increased formation of membrane structures within cells may partially contribute to the elevated levels of certain FAs observed in the livers of mice expressing the mHTT. While some differences in hepatic FA profiles were noted between the *R6/2* and *Hdh^Q150/Q150^* strains—one of which reached statistical significance—these variations are likely influenced by the distinct genetic modifications underlying each HD model. To our knowledge, the relationship between these specific genetic backgrounds and liver FA composition has not been previously explored, and the current study offers an initial step toward addressing this gap.

**Figure 4:**
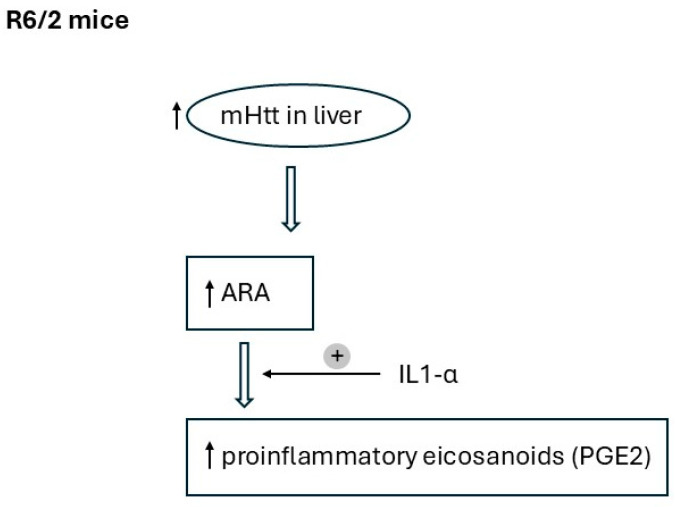
Potential mechanism of changes in FA profile on the inflammatory process in liver of *R6/2* mice; mHtt- mutant huntingtin, ↑—higher amount.

Studies indicate the influence of mHTT expression on immune activation in the brain, which plays a role in the pathogenesis of HD [22,23]. Elevated plasma levels of IL-4, IL-5, IL-6, IL-8, and IL-10 have been observed in patients with HD [22,34,35]. IL-1β level was increased in the serum of *R6/2* mice, which have an early onset of disease corresponding to the early onset of disease in humans, and did not change in *Hdh^Q150/Q150^* mice, which have a late onset of disease, compared to *WT* [36]. IL-6 levels were also elevated in the serum of *R6/2* mice, but also in *Hdh^Q150/Q150^* mice [36]. There is a relationship between inflammatory factors such as prostaglandin E2 through stimulation with TNFα and IL-1α in astrocytes this may be caused by conversion of ARA to prostaglandin E2 [37]. There are also findings showing increased TNF-α, IL-1β in the liver, cortex, striatum, and serum of *Hdh* mice and TNF-α in the liver, cortex, and serum of *R6/2* mice [29]. TNF-α concentration increased twofold in the liver of *R6/2* mice [29], as in our research IL-1α. IL-1α may also be specific for triggering pro-inflammatory changes in the liver. Inflammation associated with hepatic steatosis, liver injury, and hepatocyte regeneration can lead to fibrosis, cirrhosis, and even hepatocellular carcinoma [38]. The presence and metabolism of PUFAs and the synthesis of PUFA-derived lipid mediators are associated with inflammation [39]. Currently, n-3 PUFAs are thought to have anti-inflammatory properties, but some n-6 PUFAs such as DGLA and AdA are also associated with an anti-inflammatory response [40]. In contrast, ARA is a precursor of proinflammatory mediators [40]. We found elevated levels of ARA as well as total n-6 PUFA in *R6/2* mice, suggesting a proinflammatory FA profile. Liver inflammation might also be confirmed by elevated liver levels of IL-1α in *R6/2* mice. The potential mechanism of how changes in FA profiles in the liver may contribute to inflammation is presented in Figure 4.

The levels of ARA and PUFA did not change significantly in *Hdh^Q150/Q150^* mice, while IL1α levels were decreased. The total amount of n-6 PUFA showed a decreasing trend in *Hdh^Q150/Q150^* mice with a significantly decreased concentration of AdA. In contrast, the concentrations of anti-inflammatory n-3 PUFA did not change significantly in both *R6/2* and *Hdh^Q150/Q150^* mice. Of note is the increase in total iso-BCFA in the liver of *R6/2* mice. An approximately twofold increase in concentration was observed in iso16:0 and iso17:0 compared to *WT*. Considering that iso-BCFA has an anti-inflammatory effect in liver cells [41], it seems that the change in the concentration of iso-BCFA is related to the activation of a compensatory mechanism in the liver of *R6/2* mice that protects against inflammation but may not have been effective due to the low concentrations of iso-BCFA. In the liver of *Hdh^Q150/Q150^* mice, we found a decreasing trend of iso-BCFAs except for iso-16:0, where an increasing trend was observed. The Supplementary Table S1 presents comparison of FA levels in liver between the R6/2 and *Hdh* mice. There are a few differences (only one significant, for ETA) probably resulting from different genetic modifications of these two models of HD (Supplementary Materials). 

The limitation of this study is a lack of the analysis of mutated *HTT* gene expression in the liver. However, due to the fact that *HTT* gene expression was confirmed in the liver of mice [42] and that the presence of the *mHTT* gene was the only variable that distinguished our HD mice from controls, we believe that the differences in FA levels observed in the liver of *R6/2* mice are the effect of HD.

In conclusion, our study demonstrates that metabolic alterations, including changes in FA composition, are markedly more pronounced in the livers of mice with early-onset HD compared to those with late-onset. These findings suggest a potential link between the altered hepatic FA profile and the development of liver inflammation. Nonetheless, further comprehensive investigations are warranted to elucidate the mechanistic basis and pathophysiological significance of these findings.

## 4. Materials and Methods

### 4.1. Animal Models

Two mouse models of HD at the symptomatic stage were used in this study: 2-month-old *R6/2* mice and 22-month-old *Hdh^Q150/Q150^* mice. The *R6/2* mice carry a transgene containing the 5′ fragment of the human *HTT* gene encoding the N-terminus of huntingtin with approximately 150 CAG repeats. They are characterized by an early onset of neurological symptoms (around 6–8 weeks of age) and rapid disease progression, with the end-stage reached by 12–14 weeks [43]. The *Hdh^Q150/Q150^* mice have approximately 150 CAG repeats knocked into the mouse *Htt* gene and exhibit late-onset, slowly progressing symptoms that more closely mimic the human disease. These mouse strains typically begin to show behavioral and molecular abnormalities from 12 to 18 months of age, with significant progression by 22–24 months, depending on CAG repeat length and genetic background [44].

These two mouse strains allow for the assessment of metabolic alterations associated with early- and late-onset forms of Huntington’s disease [43,44]. Wild-type (*WT*) mice of the *C57BL/6* strain were used as controls. The experimental groups consisted of 6 *WT* mice and 5 mice in each of the *R6/2* and *Hdh^Q150/Q150^* groups.

Animals were housed in polycarbonate cages under controlled environmental conditions: a 12:12 h light/dark cycle (lights on at 06:00), constant temperature (22 °C), relative humidity of 50–55%, and ad libitum access to standard pellet food and water.

All procedures were conducted under a project license from the Home Office, UK, and approved by the ethical committee at Imperial College London and the Medical University of Gdansk Ethics Committee for Animal Experiments.

### 4.2. Analysis of the Fatty Acids

The first step of FA determination was the extraction of total lipids according to the Folch method [45] with a 2:1 (*v*/*v*) chloroform-methanol mixture and subsequent hydrolysis of the lipids with KOH in methanol. Total lipids were quantified by weighting lipids extracted by the Folch method. To obtain FA methyl esters (FAMEs), a 10% boron trifluoride-methanol solution was used. Free FAs are also present in lipid samples extracted by the Folch method, and they are derivatized to FAME in the same way as FAs released from complex lipids by hydrolysis. FAMEs were analyzed by gas chromatography–mass spectrometry (GC-MS) QP-2020 NX (Shimadzu, Shimadzu Corporation, Kyoto, Japan) [46]. FAs were identified by manual integration using FA reference standards (Larodan, Solna, Sweden, and Sigma-Aldrich, Schnelldorf, Germany). The results are presented as a mg/g of wet tissue. Individual FA content was calculated based on the peak areas of individual FA and internal standard (19-methylarachidic acid).

### 4.3. Determination of IL-1α Level

IL-1α concentration was measured in the liver using a commercially available ELISA kit according to instructions (catalog number A76782; Antibodies, St. Louis, MO, USA). The ELISA kit was a sandwich enzyme immunoassay for the quantitative measurement of IL-1α in tissue homogenates. Tissue was homogenized in PBS (1:9). The results were read against the prepared standard curve by spectrophotometry at a wavelength of 450 nm.

### 4.4. Statistical Analysis

The significance of the differences was tested using the Student *t*-test. The differences were considered significant at *p* < 0.05. The results are given as mean ± standard deviation. Statistical calculations were performed using the programs Excel and SigmaPlot 11 (Systat Software, San Jose, CA, USA).

## Figures and Tables

**Table 1:** Fatty acid concentration (µg/g of tissue) in *R6/2* and *WT* mice liver.

LIVER FAs			
	*WT*_R6/2_	*R6/2*	*p*
iso 14:0	0.002 ± 0.003	0.003 ± 0.004	NS
iso 15:0	0.006 ± 0.005	0.009 ± 0.002	NS
iso 16:0	0.017 ± 0.008	0.034 ± 0.010	<0.05
iso 17:0	0.023 ± 0.010	0.042 ± 0.008	<0.01
Total iso BCFA	0.048 ± 0.023	0.088 ± 0.012	<0.01
anteiso 15:0	0.011 ± 0.012	0.007 ± 0.004	NS
anteiso 17:0	0.009 ± 0.006	0.013 ± 0.003	NS
anteiso 19:0	0.022 ± 0.009	0.045 ± 0.012	<0.05
Total anteiso BCFA	0.042 ± 0.027	0.065 ± 0.012	NS
Total BCFA	0.090 ± 0.048	0.154 ± 0.019	<0.05
16:0	11.5 ± 4.17	17.0 ± 4.85	NS
18:0	4.65 ± 0.99	9.96 ± 3.53	<0.05
Other ECFA	0.49 ± 0.27	0.58 ± 0.16	NS
Total ECFA	16.6 ± 5.19	27.5 ± 8.44	NS
Total OCFA	0.25 ± 0.11	0.42 ± 0.13	NS
14:1	0.008 ± 0.008	0.011 ± 0.005	NS
16:1	1.46 ± 0.96	2.01 ± 0.40	NS
18:1	10.5 ± 6.75	25.0 ± 6.67	<0.05
19:1	0.012 ± 0.006	0.035 ± 0.014	<0.05
20:1	0.20 ± 0.12	0.54 ± 0.11	<0.05
22:1	0.024 ± 0.007	0.026 ± 0.009	NS
24:1	0.053 ± 0.017	0.097 ± 0.038	NS
Total MUFA	12.3 ± 7.85	27.7 ± 7.18	<0.05
18:3n-3	0.062 ± 0.021	0.072 ± 0.022	NS
20:4n-3	0.020 ± 0.009	0.066 ± 0.008	<0.01
20:5n-3	0.28 ± 0.10	0.61 ± 0.20	<0.05
22:5n-3	0.32 ± 0.11	0.44 ± 0.18	NS
22:6n-3	3.36 ± 0.77	5.19 ± 1.90	NS
Total PUFAn-3	4.05 ± 1.00	6.37 ± 2.25	NS
16:2n-6	0.014 ± 0.008	0.017 ± 0.05	NS
18:2n-6	8.89 ± 3.00	14.60 ± 3.61	<0.05
20:2n-6	0.16 ± 0.064	0.30 ± 0.057	<0.01
20:3n-6	0.57 ± 0.19	0.86 ± 0.18	<0.05
20:4n-6	3.68 ± 0.79	6.23 ± 1.66	<0.05
22:4n-6	0.093 ± 0.027	0.164 ± 0.050	<0.05
22:5n-6	0.028 ± 0.016	0.047 ± 0.013	NS
Total PUFAn-6	13.6 ± 4.07	22.5 ± 5.39	<0.05

*p* from *t*-test; values are mean ± SD; NS—nonsignificant; BCFA—branched-chain fatty acids, MUFA—monounsaturated fatty acids, PUFA—polyunsaturated fatty acids, 18:3n-3—ALA; 20:4n-3—ETA; 20:5n-3—EPA; 22:5n-3—DPA n-3; 22:6n-3—DHA; 16:2n-6—HDA; 18:2n-6—LA; 20:2n-6—EDA; 20:3n-6—DGLA; 20:4n-6—ARA; 22:4n-6—AdA; 22:5n-6—DPA n-6.

**Table 2:** Fatty acid concentration (µg/g of tissue) in *Hdh^Q150/Q150^* and *WT* mice liver.

LIVER FAs			
	*WT*_Hdh_	*Hdh^Q150/Q150^*	*p*
iso 14:0	0.009 ± 0.007	0.004 ± 0.003	NS
iso 15:0	0.012 ± 0.14	0.008 ± 0.005	NS
iso 16:0	0.04 ± 0.025	0.046 ± 0.030	NS
iso 17:0	0.045 ± 0.021	0.038 ± 0.012	NS
Total isoBCFA	0.11 ± 0.06	0.096 ± 0.041	NS
anteiso 15:0	0.024 ± 0.019	0.011 ± 0.004	NS
anteiso 17:0	0.025 ± 0.012	0.014 ± 0.01	NS
anteiso 19:0	0.064 ± 0.03	0.081 ± 0.100	NS
Total anteiso BCFA	0.11 ± 0.06	0.11 ± 0.11	NS
Total BCFA	0.22 ± 0.11	0.20 ± 0.16	NS
16:0	29.1 ± 12.9	21.5 ± 6.24	NS
18:0	12.5 ± 5.14	7.86 ± 0.74	NS
Other ECFA	0.87 ± 0.50	0.99 ± 0.64	NS
Total ECFA	42.5 ± 18.4	30.4 ± 6.95	NS
Total OCFA	0.67 ± 0.30	0.50 ± 0.14	NS
14:1	0.031 ± 0.037	0.015 ± 0.005	NS
16:1	2.62 ± 1.43	2.66 ± 2.08	NS
18:1	26.4 ± 14.4	24.7 ± 23.2	NS
19:1	0.021 ± 0.010	0.021 ± 0.024	NS
20:1	0.37 ± 0.18	0.71 ± 0.79	NS
22:1	0.032 ± 0.018	0.035 ± 0.009	NS
24:1	0.11 ± 0.036	0.07 ± 0.01	NS
Total MUFA	29.6 ± 15.9	28.2 ± 26.0	NS
18:3n-3	0.20 ± 0.09	0.10 ± 0.07	NS
20:4n-3	0.06 ± 0.05	0.03 ± 0.008	NS
20:5n-3	0.79 ± 0.32	0.38 ± 0.14	<0.05
22:5n-3	0.96 ± 0.40	0.44 ± 0.15	<0.05
22:6n-3	8.47 ± 3.45	5.08 ± 1.82	NS
Total PUFAn-3	10.5 ± 4.20	6.03 ± 2.14	NS
16:2n-6	0.04 ± 0.032	0.04 ± 0.025	NS
18:2n-6	24.2 ± 10.8	20.9 ± 13.1	NS
20:2n-6	0.39 ± 0.15	0.30 ± 0.10	NS
20:3n-6	1.01 ± 0.42	0.79 ± 0.21	NS
20:4n-6	8.31 ± 3.52	5.63 ± 1.79	NS
22:4n-6	0.30 ± 0.11	0.16 ± 0.02	<0.05
22:5n-6	0.060 ± 0.029	0.040 ± 0.011	NS
Total PUFAn-6	34.7 ± 14.4	28.1 ± 11.5	NS

*p* from *t*-test; values are mean ± SD; NS—nonsignificant; BCFA—branched-chain fatty acids, MUFA—monounsaturated fatty acids, PUFA—polyunsaturated fatty acids, 18:3n-3—ALA; 20:4n-3—ETA; 20:5n-3—EPA; 22:5n-3—DPA n-3; 22:6n-3—DHA; 16:2n-6—HDA; 18:2n-6—LA; 20:2n-6—EDA; 20:3n-6—DGLA; 20:4n-6—ARA; 22:4n-6—AdA; 22:5n-6—DPA n-6.

## Data Availability

The datasets and materials used and/or analyzed during the current study are available from the corresponding author upon reasonable request.

## Supplementary Materials

The following supporting information can be downloaded at https://www.mdpi.com/article/10.3390/ijms26157304/s1.
