# Supplementary material for: Fatty Acid Profile in the Liver of Mice with Early- and Late-Onset Forms of Huntington’s Disease
Source: Int J Mol Sci. 2025 Jul 28;26(15):7304. doi: 10.3390/ijms26157304 (PMC12347809; doi:10.3390/ijms26157304)
Supplement: Supplementary file 1 [file ijms-26-07304-s001.zip › ijms-3734240-supplementary.pdf]

Supplementary Table

Table S1. Fatty acids concentration (μg/g of tissue) in *R6/2* and *Hdh*$^{Q150/Q150}$ mice liver.

LIVER FAs			
	R6/2	*Hdh* $^{Q150/Q150}$	p
iso 14:0	0.003±0.004	0.004±0.003	NS
iso 15:0	0.009±0.002	0.008±0.006	NS
iso 16:0	0.034±0.01	0.046±0.03	NS
iso 17:0	0.042±0.008	0.038±0.012	NS
Total iso BCFA	0.088±0.012	0.096±0.04	NS
anteiso 15:0	0.007±0.004	0.011±0.004	NS
anteiso 17:0	0.013±0.003	0.014±0.01	NS
anteiso 19:0	0.05±0.01	0.08±0.1	NS
Total anteiso BCFA	0.065±0.012	0.11±0.11	NS
Total BCFA	0.15±0.02	0.20±0.15	NS
16:0	16.9±4.9	21.5±6.2	NS
18:0	9.9±3.5	7.8±0.74	NS
Other ECFA	0.6±0.2	0.99±0.64	NS
Total ECFA	27.5±8.4	30.4±6.9	NS
Total OCFA	0.42±0.13	0.49±0.14	NS
14:1	0.01±0.005	0.02±0.01	NS
16:1	2.0±0.4	2.7±2.1	NS
18:1	24.9±6.7	24.7±23.2	NS
19:1	0.04±0.01	0.02±0.02	NS
20:1	0.54±0.11	0.71±0.76	NS
22:1	0.026±0.01	0.035±0.01	NS
24:1	0.1±0.04	0.07±0.01	NS
Total MUFA	27.98±7.2	28.2±26.0	NS
18:3n-3	0.07±0.02	0.10±0.07	NS
20:4n-3	0.07±0.01	0.03±0.01	<0.05
20:5n-3	0.6±0.2	0.38±0.14	NS
22:5n-3	0.44±0.18	0.44±0.14	NS
22:6n-3	5.2±1.9	5.1±1.8	NS
Total PUFAn-3	6.3±2.2	6.0±2.1	NS
16:2n-6	0.02±0.01	0.04±0.03	NS
18:2n-6	14.6±3.6	20.9±13.1	NS
20:2n-6	0.3±0.1	0.3±0.1	NS
20:3n-6	0.86±0.18	0.79±0.2	NS
20:4n-6	6.2±1.7	5.6±1.8	NS
22:4n-6	0.16±0.05	0.16±0.02	NS
22:5n-6	0.05±0.01	0.04±0.01	NS
Total PUFAn-6	22.5±5.4	28.2±11.5	NS

p from *t*-test; values are mean±SD; NS - nonsignificant; BCFA- branched-chain fatty acids, MUFA- monounsaturated fatty acids, PUFA- polyunsaturated fatty acids, 18:3n-3 – ALA; 20:4n-3 – ETA; 20:5n-3 - EPA; 22:5n-3 – DPA n-3; 22:6n-3 – DHA; 16:2n-6 -HDA; 18:2n-6 – LA; 20:2n-6 -EDA; 20:3n-6 – DGLA; 20:4n-6 – ARA; 22:4n-6 – AdA; 22:5n-6 – DPA n-6.
